# Methods and approaches to facilitate inclusion of the views, perspectives and preferences of people with moderate‐to‐severe dementia in research: A narrative systematic review

**DOI:** 10.1111/opn.12594

**Published:** 2023-12-11

**Authors:** Rachel Collins, Anthony Martyr, Anna Hunt, Catherine Quinn, Claire Pentecost, Julian C. Hughes, Linda Clare

**Affiliations:** ^1^ Centre for Research in Ageing and Cognitive Health University of Exeter Medical School Exeter UK; ^2^ Centre for Applied Dementia Studies University of Bradford Bradford UK; ^3^ Wolfson Centre for Applied Health Research Bradford UK; ^4^ Population Health Science Institute, Bristol Medical School, University of Bristol Bristol UK; ^5^ NIHR Applied Research Collaboration South West Peninsula Exeter UK

**Keywords:** Alzheimer's disease, communication, conversation, interview

## Abstract

**Background:**

The perspectives of people with moderate‐to‐severe dementia are rarely directly elicited in research studies.

**Objectives:**

This systematic review will explore methods and approaches for including the perspectives and preferences of people with moderate‐to‐severe dementia in research.

**Methods:**

AgeLine, CINAHL, Embase, PsycINFO, PubMed, Social Policy and Practice and Web of Science were searched until June 16 2022. Study quality was assessed using the 16‐item Quality Assessment Tool. We described specific communication tools, reviewed the evidence for their effectiveness and considered their strengths and limitations. We examined the more general communication skills and techniques applied to support the use of these tools using thematic synthesis. The review protocol was registered with PROSPERO CRD42019130386 and the review was conducted and reported according to PRISMA guidelines.

**Results:**

Seven studies reported in 11 publications were included. In these studies five specific communication tools were used: Talking Mats, Augmentative and Alternative Communication Flexiboard, generic photographs in combination with a preference placement board, consultation ballot and personalised communication prescriptions. Each tool identified had advantages and disadvantages depending on dementia severity, verbal or physical ability, expense, researcher training requirements and ease of use. Thematic synthesis identified five general approaches to optimising communication that were employed to support use of the tools: ensuring conversations are individual and person‐centred, managing external influences, engaging others, creating structure and facilitation skills.

**Conclusion:**

All tools had some utility and there was no clear evidence to support the recommendation of any one specific tool; therefore, researchers are advised to select the tool most appropriate to their context.

**Implications for Practice:**

The findings offer general guidance for researchers and practitioners on how to facilitate communication with people with moderate‐to‐severe dementia.

**What does this research add to existing knowledge in gerontology?**

- The review identifies the key communication skills and methods that practitioners and researchers can use to facilitate meaningful conversations with people who have moderate‐to‐severe dementia.
- The review outlines the evidence‐base for selecting structured communication tools that can help with eliciting the views and preferences of people with moderate‐to‐severe dementia.

**What are the implications of this new knowledge for nursing care with older people?**

- The review offers evidence‐based guidance for practitioners and researchers on how to communicate effectively with people who have moderate‐to‐severe dementia.
- The structured communication tools reviewed all have strengths and limitations; therefore, rather than recommending one tool, practitioners and researchers are advised to select the tool best suited to their own context.

**How could the findings be used to influence policy or practice or research or education?**

- The findings provide guidance on best practice for nursing staff, other practitioners and researchers wishing to elicit the opinions and preferences of people with moderate‐to‐severe dementia.
- The findings could be used to help increase involvement of people with moderate‐to‐severe dementia in research and in decisions about their lives and care.

## 1 INTRODUCTION

Over 57 million people worldwide are living with dementia (Nichols et al., 2022). To support those diagnosed with dementia to ‘live well’ with the condition it is important to understand their individual experiences, preferences and care needs (Poulos et al., 2017). The concept of ‘living well’ is defined as ‘the best achievable state of health that encompasses all dimensions of physical, mental and social well‐being’, reflected in ‘a self‐perceived level of comfort, function, and contentment with life’ (The Institute of Medicine, 2012, p. 32). To understand whether someone is living well, and identify the impact of care, services or interventions on a person's capability to live well, it is important to obtain the person's perspective and opinions. It is generally accepted that people with mild‐to‐moderate dementia can provide reliable responses to standardised measures (Clark et al., 2008) as well as giving rich accounts in detailed interviews, but people whose dementia has progressed further are less well represented in research (Hoe et al., 2005). Often, opinions of informal carers are sought and informant ratings are substituted for self‐ratings, but this approach has its limitations as these are not equivalent and reliability of informant accounts can vary (Banerjee et al., 2009; Lacey et al., 2015; Martyr & Clare, 2018; Moyle et al., 2012; Torisson et al., 2016; Wu et al., 2020).

Directly obtaining the perspectives of people with moderate‐to‐severe dementia in research studies is preferable but requires more effort and a different approach. Verbal communication to describe feelings, preferences or decisions may become more limited as dementia progresses, requiring additional support (Bilodeau et al., 2019; Schrauf, 2020; Wray, 2020), while non‐verbal communication is often relatively preserved (Clare, Quinn et al., 2014; Hughes, 2013; Quinn et al., 2014; Round et al., 2014). Augmenting communication and using a combination of verbal and non‐verbal elements may enable the person to provide responses or indicate preferences (Clare et al., 2008a; Moore & Hollett, 2003), especially where there is judicious use of tools and techniques designed to facilitate both elements (Alsawy et al., 2017). Understanding more about this would not only be valuable for researchers but would also benefit practitioners.

In the context of the IDEAL (Improving the experience of Dementia and Enhancing Active Life) cohort study investigating ‘living well’ among people with mild‐to‐moderate dementia in the community (Clare, Nelis et al., 2014; Clare et al., 2019) where researchers directly elicited participants' views through standardised questionnaires and open‐ended questions, we wanted to explore ways of including people with more advanced, moderate‐to‐severe dementia who can still communicate verbally but who find standardised questionnaires too challenging and need a different approach to enable them to contribute their views. As there was no review on the topic, we set out to identify the available methods, tools and techniques that have been employed in research studies to elicit the views, perspectives and preferences of people living with moderate‐to‐severe dementia who are still able to communicate verbally, and to evaluate the utility of the identified methods and approaches.

## 2 METHODS

This narrative systematic review followed PRISMA reporting guidelines (Page et al., 2021).

### 2.1 Search strategy

The following databases were searched: AgeLine, CINAHL, Embase, PsycINFO, PubMed, Social Policy and Practice and Web of Science. The search terms were (dementia or Alzheimer* or vascular dementia or Lewy body or frontotemporal) AND (nonverbal OR communication OR communicate OR language OR speech OR speak OR voice OR talk* OR Talking Mats OR Augment* Alternative OR AAC); see the Data S1 for the search strings used in each database. There were no restrictions on the date of publication, and hence no start dates were applied to the searches. Initial searches took place on April 17, 2019 and the searches were updated on June 16, 2022. The protocol was registered with PROSPERO: CRD42019130386.

### 2.2 Inclusion and exclusion criteria

Studies describing communication methods for, or using communication methods with, people with moderate‐to‐severe dementia living either in the community or in residential care were identified. All study types were potentially eligible, but only texts available in English were included. Initial scoping work suggested that evidence was limited. Therefore, we decided to include reports in addition to peer‐reviewed journal articles, and to include studies irrespective of quality ratings. Eligible studies had to demonstrate or describe methods to enable people with moderate‐to‐severe dementia who were still able to communicate verbally to express their views and preferences as part of a research study or as part of the research process. They also had to include methods that researchers or practitioners could potentially use, for example tools, techniques, structured or unstructured activities, directed or undirected communication or psychosocial interventions.

### 2.3 Screening and selection

Title, abstract and full‐text screening was undertaken independently by two researchers. Any disagreements over inclusion were discussed with a third researcher and a consensus achieved. Where data from the same study were reported across several publications, the details were considered together to avoid dual counting or repetition. Reference lists of articles that met full text screening criteria were screened for additional articles.

### 2.4 Appraisal of study quality

Study quality was independently assessed by two researchers using the 16‐item Quality Assessment Tool (QATSDD; Sirriyeh et al., 2012). Each item was scored from 0 (criterion undescribed) to 3 (criterion described in full). For each study the item scores were summed to provide a total score out of 48, which was converted to a percentage. The higher the score, the greater the quality of the study. As the QATSDD does not offer criteria for grouping studies into categories reflecting different levels of quality, three score bands were created reflecting poor (≤49%), moderate (50%–79%) and high (≥80%) quality.

### 2.5 Data extraction and analysis

Characteristics of the included studies, details of the tools and communication methods used, and data regarding the effectiveness of these tools and methods in facilitating communication with people who have moderate‐to‐severe dementia were extracted by two independent researchers using a pre‐designed form. The two sets of extracted data were compared and checked for consistency by a third researcher. We summarised and reviewed the descriptions of the tools, their strengths and limitations and the evidence presented regarding effectiveness. The text of all the included studies was then imported into NVivo v12 and subjected to thematic synthesis following the first two steps outlined by Thomas and Harden (2008). We coded author statements and, where available, direct participant quotes referencing skills or techniques that facilitated either the use of specific communication tools or communication more generally. In the first step, two researchers independently coded the methods, results, and discussion sections of each paper. A codebook was developed and refined through an iterative process whereby new codes were added, and existing codes altered or merged, as successive articles were analysed. In the second stage, the codes were grouped according to conceptual similarity and groups were combined into descriptive themes reflecting techniques for facilitating communication. The themes were refined through discussion with the wider research team. The results are presented in narrative form.

## 3 RESULTS

**FIGURE 1:**
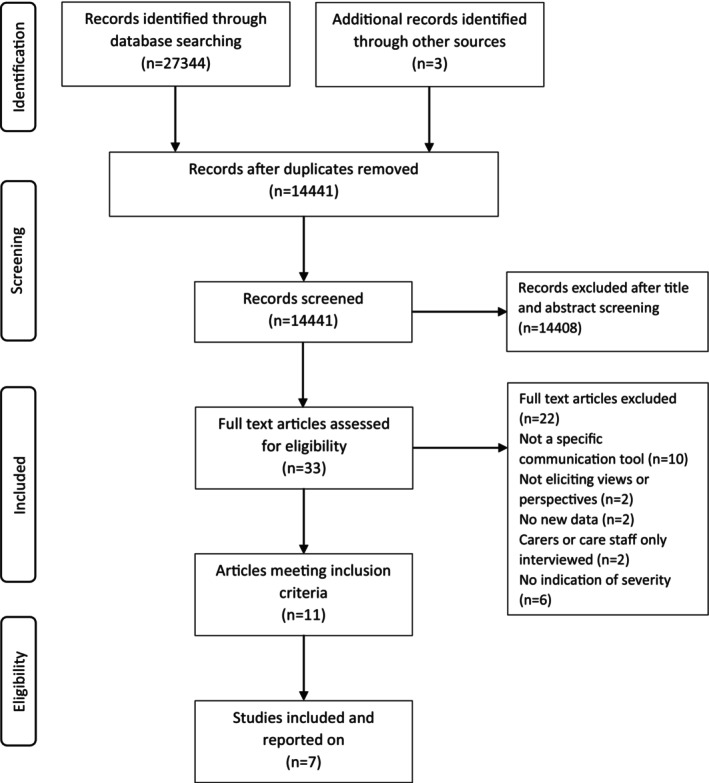
Flow diagram illustrating the study selection process.

Eight journal articles (Acton et al., 2007; Burshnic & Bourgeois, 2022; Fried‐Oken et al., 2012; Godwin, 2014; Murphy et al., 2007b; Murphy, Gray, et al., 2010; Murphy & Oliver, 2013; Oliver et al., 2010) and three reports (Murphy et al., 2007a; Murphy et al., 2010; Williamson, 2010) were included in the review; see Figure 1. These 11 documents reported data from seven discrete research studies. Five studies were each described in one single publication, and two studies were each described in two journal articles and an extended report, one in Murphy et al. (2007a, 2007b) and Murphy, Gray, et al. (2010) and the other in Murphy and Oliver (2013), Murphy, Oliver, and Cox (2010) and Oliver et al. (2010). Table S1 lists the studies excluded at full‐text screening with reasons for exclusion.

### 3.1 Study characteristics

See Table 1 for a summary of study characteristics. All the studies were conducted in the United States or the United Kingdom, and all used both quantitative and qualitative methods. Sample sizes were small, ranging from 10 to 34. The included studies overall provided information from 177 people with dementia and 60 carers (both paid carers and family members). Most of the people with dementia (61.0%) and carers (88.3%) were female. Ages of the people with dementia were given in five cases and ranged from 50 to 103 years. Information about stage of dementia was inconsistently reported. Where objective measures were provided and stage categorised (Acton et al., 2007; Burshnic & Bourgeois, 2022; Murphy et al., 2007a; Murphy et al., 2010), 32 people were described as having ‘moderate’ and 34 as having ‘severe’ dementia. Studies using subjective evaluations stated that 11 people had ‘moderate’ and 44 had ‘severe’ or ‘advanced’ dementia (Godwin, 2014; Murphy et al., 2007a). One study reported in three publications (Murphy & Oliver, 2013; Murphy et al., 2010; Oliver et al., 2010) included people living in the community only, two (Acton et al., 2007; Godwin, 2014) included people living in residential care only and the remaining four studies included people from both settings.

**TABLE 1:** Characteristics of the included studies.

Study	Location	Participant characteristics			Living situation	Study quality rating
		Demographics	Diagnosis	Severity		
Acton et al. (2007)	USA	*n* = 10 Sex: M (1); F (9) Age: Mean 81 years (76–88)	Dementia	MMSE 2–25; 2 mild, 6 moderate, 2 severe	Residential care home	Moderate
Burshnic and Bourgeois (2022)	USA	*n* = 21 Sex: M (0); Female (21) Age: Mean 88.9 ± 7.55 (77–103)	19 (90.4%) Unspecified dementia 2 (9.5%) Alzheimer's disease	Severe BIMS 4 ± 2.00 (0–7)	Residential care home Assisted living facilities	Moderate
Fried‐Oken et al. (2012)	USA	Pilot 1: *n* = 30 Sex: M (7); F (23) Age: Mean 74 years (50–94) Pilot 2: *n* = 11 Sex: M (3); F (8) Age: Mean 73 years (60–85)	Alzheimer's disease	MMSE 5–18 (Mean 12)	Private family home Residential care home	Moderate
Godwin (2014)	UK	PwD: *n* = 34 Sex: M (5); F (29) Age not stated Staff: *n* = 42 Sex: M (2); F (40)	Dementia including dementia with Lewy bodies	Advanced (no objective scores provided)	Residential care home (Dementia specific)	Poor
Murphy, Gray, et al. (2010)	UK	*n* = 31 Age: Range 54–90	Not reported, but as below.	Originally assessed by staff: 10 ‘early‐stage dementia’, 11 ‘moderate stage dementia’, 10 ‘late stage dementia’	Private family home Residential care home Sheltered housing with warden	Moderate
Murphy et al. (2007a) reporting on the same study as Murphy, Gray, et al. (2010)	As above	As above, plus: Sex: M (9); F (22)	23% mixed‐type dementia 19% Alzheimer's disease 10% multi‐infarct dementia 6% vascular dementia 6% Korsakoff's syndrome 35% unspecified	As above, plus: Subsequent CDS ratings: 9 early, 13 moderate, 9 late	As above	As above
Murphy et al. (2007b) reporting on the same study as Murphy, Gray, et al. (2010)	As above	As above	As above	As above	As above	As above
Murphy and Oliver (2013)	UK	PwD: *n* = 18 Sex: M (10); F (8) Age: Mean 77 years (60–86) Carer: *n* = 18 Sex: M (5); F (13) Age: 69 years (44–89)	Dementia	3 early, 13 moderate, 2 late stage	Private family home	Moderate
Murphy, Oliver, and Cox (2010) reporting on the same study as Murphy and Oliver (2013)	As above	As above	As above	As above, plus: Severity stages were determined by scores on the CDS.	As above	As above
Oliver et al. (2010) reporting on the same study as Murphy and Oliver (2013)	As above	As above	As above	As above	As above	As above
Williamson (2010)*	UK	*n* = 22 Sex: M (13); F (9) Age: Not stated	Not stated	Subjectively described	Residential care homes Private family home	Moderate

*Note*: The CDS rating was developed during the project but the original staff ratings were used in the data analysis.

Abbreviations: BIMS, Brief Interview for Mental Status; CDS, Communication Difficulties Scale (Murphy et al., 2007a); MMSE, Mini‐Mental State Examination; PwD, people with dementia.

* This study also included a postal survey and focus group. No details of participants or outcomes specifically with respect to dementia severity were presented; consequently, no information from this part of the study was included.

### 3.2 Study quality

All but one of the studies were rated as being of moderate quality. One (Godwin, 2014) fell into the poor quality score band mainly due to providing a less detailed account of the methodological approach. All studies adequately described the aims, research setting and data collection procedures and used a method that fitted the research question. However, all studies had small, unrepresentative samples, and where quantitative approaches and statistical analyses were used there was no consideration of the implications of small sample sizes. Only two studies (Acton et al., 2007; Godwin, 2014) drew on an explicit theoretical framework. We did not exclude any study based on quality. See Table S2 for QATSDD scores (Sirriyeh et al., 2012).

### 3.3 Specific tools and methods used to facilitate communication

Three specific approaches based on the use of pictures or symbols to facilitate conversations were identified: Talking Mats, Augmentative and Alternative Communication (AAC) Flexiboard and a preference sorting template. Table 2 summarises findings about effectiveness of the tools.

**TABLE 2:** Quantitative outcomes reported in the included studies.

Study	Communication method	Evaluation of data	Summary of results	
			Primary outcome	Improvement in communication outcomes
Acton et al. (2007)	Two interviews: Interview 1—15 min interview analysed to develop an individualised communication prescription. Interview 2—using the individualised prescription generated from first interview	Differences between Interview 1 and 2 total number of participants’ words, average of participants’ words per topic total number of topics needed to sustain the interview between interview 1 and interview 2.	*All participants*:	
			Total number of words	No
			Average words per topic	Yes (*p* < .05)
			Total number of topics	Yes (*p* < .05)
			Difference by low (2–5), medium (14–19), or high (24–25) MMSE scores	No
			*Participants with low MMSE scores* (*n=2*):	
			Total number of words	Yes
			Average number of words per topic	Yes
			Percentage of topics	Yes
			Total number of topics	Yes
Burshnic and Bourgeois (2022)	Two interviews using cross‐over method 1 week apart. Participants were asked about preferences using two sets of preference questions (Based on MDS 3.0 and PELI‐NH). Standard format used preference rating template only; Supported condition used preference rating template plus printed photographs accompanied by textual captions	Preference consistency (very, somewhat, or not important) in response to preference questions (e.g., How important is it to you to exercise?) between standard vs supported format (1 week apart) Differences in utterance type between standard vs supported format (1 week apart) Procedural fidelity	Effect of condition (supported vs. standard)	
			Preference consistency	No
			*Utterance type*	
			Asking for clarification	Yes (*p* < .01)
			Acknowledgements (e.g., “I enjoy it” rather than a preference	No
			Elaboration by participant (e.g., goes into a related story)	No
			Off‐topic utterances	No
			*Procedural fidelity*	No
Fried‐Oken et al. (2012)	*Pilot 1*—10‐min conversations with and without personalised AAC boards *Pilot 2*—Participants were randomly assigned to one of the six symbol‐type/voice output conditions (print alone, 2D symbols + print, 3‐D symbols + print; all with and without voice output)	Independent variables—AAC support, symbol type, and voice output Social communication framework *Pilot 1*: Dependent variables: Number of utterances Percent of flag collateral (percent of all utterances that included flag collateral) Percent of explanatory collateral (percent of all utterances that included explanatory collateral) Percent of one‐word utterances Number of references to AAC device (if present) *Pilot 2*: Dependent variables: Number of targeted words used Percent of targeted words used Percent of related words	*Pilot 1*	
			Across the five dependent variables	
			AAC support vs No AAC	No
			Different symbol types (print, 2D+print, 3D+print)	No
			Use of voice output used with AAC	Yes (*p* < .010)
			*Pilot 2*	
			AAC support evaluated by comparing control, primed control, primed AAC (pairwise comparisons)	
			*No. Targeted words*	
			Primed AAC vs control	Yes (*p* < .029)
			Primed AAC vs primed control	Yes (*p* = .032)
			Control vs primed control	No
			*Percent of targeted words*	
			The percent of targeted words was significant	Yes (*p* < .024)
			Primed AAC vs control	Yes (*p* < .013)
			Primed AAC vs primed control	Yes (*p* < .027)
			Control vs primed control	No
			*Percent of all related words*:	
			The effect of condition	No
Godwin (2014)	Consultation with every resident to seek their colour preferences using a ballot.	Number of votes for each colour choice	Residents’ votes: Blue 10; Red 5; Yellow 4.75; Mauve 3.75; Orange 3.5 Staff votes: Mauve 8.5; Orange 4.83; Yellow 2.83; Blue 1; Red 0.83 Total: Red 5.8; Orange 8.3; Yellow 7.6; Blue 11.0; Mauve 12.25; Don't mind 2.0; Total 47.0	Yes (Participants made a choice)
Murphy, Gray, et al. (2010)	4 topics (activities, people, environment, self) were discussed under 3 interview conditions: (1) Unstructured Conversation: the researcher asked participants to tell her about each topic. (2) Structured conversation: different options for each topic were discussed in turn (3) Talking Mats: visual symbols representing the topics and options were created and participants were asked to place these under a visual scale to indicate their feelings (e.g., happy, not unsure, unhappy)	Effectiveness framework of functional communication. The video recordings of the interviews were coded; the following four communication indicators were rated on a 5‐point scale from 0(never) to 4 (always): The participant's understanding of the options presented – based on verbal and non‐verbal responses perseveration Engagement of the participant with the interviewer and the task The amount of time during the interview that the content of the participant's communication was ‘on track’ the interviewer's understanding of the participant's views b instances of perseveration across the 3 interview conditions. c Proportion of time spend engaged in ‘on‐task’ behaviours for each of the 3 interview conditions. d Time taken for each of the 3 interview conditions.	Overall communication effectiveness scores:	
			*Moderate‐stage*:	
			Talking Mats vs Structured Conversation	Yes (*p* < .01)
			Talking Mats vs Unstructured Conversation	Yes (*p* < .01)
			*Late‐stage* ^a^	
			Talking Mats vs Structured Conversation	Yes (*p* < .05)
			Talking Mats vs Unstructured Conversation	Yes (*p* < .01)
			b Instances of perseveration	
			*Moderate‐stage*:	
			Talking Mats vs Structured Conversation	Yes (*p* < .05)
			Talking Mats vs Unstructured Conversation	Yes (*p* < .05)
			*Late‐stage* ^a^	
			Talking Mats vs Structured Conversation	Yes (*p* < .05)
			Talking Mats vs Unstructured Conversation	Yes (*p* < .05)
			c ‘On‐task’ behaviours:	
			Moderate‐stage:	
			Talking Mats vs Structured Conversation	Yes (*p* < .05)
			Talking Mats vs Unstructured Conversation	No
			*Late‐stage* ^a^	
			Talking Mats vs Structured Conversation	Yes (*p* < .05)
			Talking Mats vs Unstructured Conversation	Yes (*p* < .05)
			d time:	
			*Moderate‐stage*:	
			Talking Mats vs Structured Conversation	No
			Talking Mats vs Unstructured Conversation	Yes (*p* < .001)
			*Late‐stage* ^a^	
			Talking Mats vs Structured Conversation	Yes (*p* < .001)
			Talking Mats vs Unstructured Conversation	Yes (*p* < .001) note: significant results indicate conversations using talking mats took longer.
Murphy et al. (2007a) reporting on the same study as Murphy, Gray, et al. (2010)	As above	As above, plus: Individual indicators within the effectiveness framework	Individual indicators within the effectiveness framework	
			Participant understanding	
			*Moderate‐stage*	
			Talking Mats vs Structured Conversation	Yes (*p* < .05)
			Talking Mats vs Unstructured Conversation	Yes (*p* < .05)
			*Late‐stage*:	
			Talking Mats vs Structured Conversation	Yes (*p* = .051)Yes (*p* = .051)
			Talking Mats vs Unstructured Conversation	Yes (*p* < .05)
			Engagement	
			*Moderate‐stage*	
			Talking Mats vs Structured Conversation	Yes (*p* < .05)
			Talking Mats vs Unstructured Conversation	Yes (*p* < .05)
			*Late‐stage*	
			Talking Mats vs Structured Conversation	Yes (*p* = .051)
			Talking Mats vs Unstructured Conversation	No
			On track	
			*Moderate‐stage*	
			Talking Mats vs Structured Conversation	Yes (*p* < .05)
			Talking Mats vs Unstructured Conversation	Yes (*p* < .05)
			*Late‐stage*:	
			Talking Mats vs Structured Conversation	Yes (*p* < .05)
			Talking Mats vs Unstructured Conversation	Yes (*p* < .05)
			Researcher understanding:	
			*Moderate‐stage*:	
			Talking Mats vs Structured Conversation	Yes (*p* < .01)
			Talking Mats vs Unstructured Conversation	Yes (*p* < .05)
			*Late‐stage*	
			Talking Mats vs Structured Conversation	Yes (*p* < .05)
			Talking Mats vs Unstructured Conversation	No^b^
Murphy et al. (2007b) reporting on the same study as Murphy, Gray, et al. (2010)	As above	As above	No quantitative data reported in this article but as above.	No quantitative data reported in this article but as above.
Murphy and Oliver (2013)	Dyads discussed four topics under two conditions. Topics: Personal care (washing, getting dressed) Getting around (walking, driving) Housework (cooking, laundry) Activities (Listening to music, reading a book). Conditions: Talking Mats: the topics and options were converted into symbols. Dyads placed these under the visual scale to indicate if the person with dementia was ‘managing’, ‘needed assistance’, or ‘not managing’. 2 Usual communication methods: the researcher presented each option verbally and couples were asked to discuss if the person with dementia was ‘managing’, ‘needed assistance’, or ‘not managing’.	Dependent variables: Level of involvement in each type of discussion. Levels of satisfaction with each type of discussion	Talking Mats vs. usual communication methods	
			Involvement in discussion	Yes (*p* < .01)
			b Satisfaction with discussion:	Yes (*p* < .01)
			Talking Mats: Involvement family carers vs people with dementia	Yes (*p* < .05)
Murphy, Oliver, and Cox (2010) reporting on the same study as Murphy and Oliver (2013)	As above	As above, plus	Talking Mats vs Usual Conversation	
		Effectiveness coding framework:	1. Effectiveness	
		(a) Participant's understanding of topic for discussion	Overall effectiveness score	Yes (*p* < .05)
		(b) Participant's engagement with process	Individual effectiveness indicators	
		(c) The amount of time during the interview that the content of the participant's communication was ‘on track’	a) Participant understanding:	No
		(d) Researcher's understanding of participant's views	b) Participant engagement:	Yes (*p* < .05)
		(e) Participant's confidence level in responding	c) Participant on track:	No
			d) Researcher understanding:	No
			e) Participant confidence in responding:	No
		(2) Additional aspects of effective communication	(2) Additional aspects of effective communication	
		Perseveration	Perseveration	Yes (*p* < .05)
Oliver et al. (2010) reporting on the same study as Murphy and Oliver (2013)	As above	As in Murphy and Oliver (2013	No quantitative data reported in this article but as in Murphy and Oliver (2013	No quantitative data reported in this article but as in Murphy and Oliver (2013.
Williamson (2010)	To determine quality of life indicators. Primary research to gather the views of people with more severe dementia using an adapted Talking Mats Postal survey Consultation with key stakeholders, including people with dementia and family carers.	Responses to Talking Mats picture card exercise and survey. Analyses interview and focus groups to identify when participants mentioned factors that affected their quality of life e.g., upsetting, enjoyment). Quality of life indicators weighted according to how important they were to the participant (content, frequency). Top 5 most frequent quality of life indicators identified as ‘very important’	Most important (top 5) quality of life indicators for those in care home (severe dementia): Nice place to live Someone to talk to Feeling fit and well Having a laugh Feeling safe and secure	Yes (quality of life indicators obtained)

Abbreviations: AAC, augmentative and alternative communication; MMSE, Mini‐Mental State Examination; MDS 3.0, minimum data set, version 3 (Saliba & Buchanan, 2008); PELI‐NH, preferences for everyday living inventory, nursing home version (Curyto et al., 2016; Van Haitsma et al., 2012).

^a^ Below the authors stated effectiveness level.

^b^ Authors state marginally significant (*p* = .066).

The Talking Mats tool was used in the two studies by Murphy and colleagues (Murphy et al., 2007a, 2007b; Murphy, Gray, et al., 2010; Murphy & Oliver, 2013; Oliver et al., 2010) and in Williamson (2010). Talking Mats involves placing picture symbols on a textured mat as a conversation progresses. Pre‐defined symbols represent topics being discussed, options related to the topic and a visual scale for the participants to indicate their feelings. A tablet version (Murphy & Ewing, 2018) is available but the report describing its use did not meet inclusion criteria. Statistical analyses suggested that Talking Mats were more effective in facilitating communication than alternative methods. Use of Talking Mats made it possible to identify important indices of quality of life among people with severe dementia living in care homes (Williamson, 2010).

AAC Flexiboard (Fried‐Oken et al., 2012) is a touch‐sensitive device that can be programmed with pictures, personal photographs, 2D and 3D symbols, digitised speech and/or vocabulary overlays. It supports use of AAC as a strategy to aid word finding and language production (Fried‐Oken et al., 2015). Improvement in communication was achieved using this tool (Fried‐Oken et al., 2012).

The third picture‐based approach (Burshnic & Bourgeois, 2022) involved using a preference sorting template to support participants in answering questions about how important various activities were to them. Participants were given a poster board containing three sorting boxes labelled ‘Very important’, ‘Somewhat important’ and ‘Not important’. For each question participants were given a card containing a photograph of the activity with a written caption describing the activity. Participants were asked to place each card in one of the three boxes to communicate their answer. This was compared with giving participants a printed list of the three sorting options and asking them to say how important the activity was. While participants were able to provide clear answers in both conditions, there were significantly fewer requests for clarification when using the preference sorting template.

Two studies used methods that were not picture‐based. One involved developing an individualised communication strategy for the person with dementia based on analysis of a preliminary interview. The communication strategy sets out the methods and techniques that facilitate or inhibit communication for the given individual (Acton et al., 2007). This yielded positive benefits, but the study involved only two people. The other non‐picture–based method involved using a consultation ballot to obtain participants' opinions (Godwin, 2014).

### 3.4 Utility of the tools

These tools and methods were assessed regarding their availability and ease of use; see the summary of advantages and disadvantages in Table 3.

**TABLE 3:** Advantages and disadvantages of the tools and methods identified in the included studies.

Study	Tool	Description	Potential advantages	Potential disadvantages
Acton et al. (2007)	Prescribed interview	Utilised preliminary interview to develop and produce an individualised communication strategy. Prescribed interview later used and communication effectiveness compared with first interview.	Person‐centred, unique for each individual More flexible with conversation, not constrained by images or symbols Lower long‐term cost implication	High level of skill required to generate a prescribed interview High level of skill required to implement and analyse prescribed interview
Burshnic and Bourgeois (2022)	Printed photographs from Google Images™ with textual captions along with printed, preference rating template	Validated printed photographs with captions were used as prompts to aid or stimulate preference rating of social contact, personal development and leisure activities. A template comprising printed text rating preferences was also used. (1 = Very important; 2 = Somewhat important; 3 = Not important)	Cost effective Easy to obtain images No staff training required Relatively easy for participants with fewer requests for clarity and less going off topic Good for one‐to‐one conversations and gaining individual preferences. Good for different levels of verbal ability including those with more severe dementia	Generic pictures may be less effective than personally relevant pictures Potentially more effective if participants are given opportunity to practice prior to obtaining preferences In this study preference ratings were limited to three options and therefore subtle preferences are likely to be missed Potentially a smaller sample could be included in the research
Fried‐Oken et al. (2012)	AAC Flexiboard	The tool uses a combination of images, photographs, and 3D symbols that are personal to the participant and obtained from family carers prior to the conversation taking place.	Effective in increasing level of conversation Stimuli used so can be tailored to individual e.g. obtained from family carers Suitable for those with visual impairments Stimuli may act as a memory aid	Cost implications Registered product Training required Participants need specific training with product to achieve effective communication Potentially a smaller sample could be included in the research Use of voice output may impede conversation
Godwin (2014)	Activity based	Different methods could be used but this example used a ballot‐based system to gauge opinion of wall colour.	Effective in obtaining opinion on a specific topicUseful for larger objective e.g. preferences and choices Accessible method for researchers and practitioners	More preparation needed to develop and design May be more resistance from residential care management and/or family carers May exclude some residents Caution required to obtain robust research outcomes Potentially more challenging for obtaining complex and subtle meaning, preferences, and emotions
Murphy, Gray, et al. (2010); Murphy et al. (2007a); Murphy et al. (2007b); Murphy and Oliver (2013); Murphy, Oliver, and Cox (2010); Oliver et al. (2010); Williamson (2010)	Talking Mats	Pre‐designed symbols representing themes or activities are placed by participants at specific points on a board or mat to indicate general and emotional feelings about a specific subject.	Effective in gauging opinion from participants Increases feelings of engagement, interaction, and satisfaction in participants Specific symbols can be created for different participants e.g. culturally appropriate Pictures may act as a memory aid	Cost implications Registered product Training required Participants need to be familiar with product to stimulate effective communication Pre‐defined symbols Symbols can be too simple to convey complex meanings and emotions Symbols can be construed as childish or inappropriate Less effective for people with severe dementia Limited to those with good eyesight and/or hand dexterity Time consuming Potentially a smaller number of participants could be included in the research Potentially more cognitively demanding than a simple conversation and regular breaks should be considered

Each of the five tools is suitable for use in one‐to‐one conversations with people with moderate‐to‐severe dementia. Talking Mats, the preference sorting template, and the ballot method were all effective in eliciting specific responses. They may therefore be more useful for research purposes than individualised communication prescriptions and the AAC Flexiboard, which aimed primarily to encourage social conversation. While the ballot method is limited to obtaining responses about one specific topic, Talking Mats, the preference sorting method and the AAC Flexiboard all allow for more nuanced responses about multiple topics.

A key strength of the preference sorting and ballot methods is accessibility; researchers can create these tools themselves at low cost. The other tools are less accessible due to the cost of purchasing the tool in the case of Talking Mats and the AAC Flexiboard, or the time and expertise needed to generate and use individualised communication prescriptions. Another strength of the preference sorting and ballot methods is that minimal training in their use is required. In contrast, training is recommended for Talking Mats and training sessions are run by the providers for additional cost. For the AAC Flexiboard, in‐house training in setting up and using the different features would be required. The tool with the highest requirement for specialist skills is the individualised communication prescription method.

While each of the tools is suitable for use with people who have moderate‐to‐severe dementia, a major strength of the three picture‐based tools is that they do not require speech or writing ability. They may also prove useful where people with dementia do not share a common language with the researcher. However, their use does require sufficient visual acuity to see the images and sufficient manual dexterity to place the symbols. Individualised communication prescriptions are suitable for people who communicate verbally, and can be used regardless of visual acuity, mobility or manual dexterity issues. The ballot method can also be easily adapted to accommodate different sensory, mobility and manual dexterity needs.

Regarding ease of use, communication using picture‐based tools takes longer than standard conversations and people with dementia may need training to become familiar with these tools. While this may be feasible in a research context, it might limit use in busy care settings. Ensuring that pictures are representative across individuals, environments and cultures is another challenge and, while personalised images can be incorporated, these are effortful to obtain. A strength of the ballot and individualised communication prescription methods is that they require no prior training for the person with dementia. However, while collating ballot materials requires relatively little effort on the part of the researcher, the opposite is true for developing and implementing individualised communication prescriptions.

### 3.5 Skills and techniques for facilitating communication

Although the included studies were diverse, thematic synthesis identified several techniques applied in the research to support the use of the specific tools. Five themes were identified, and these are described below; see Table 4 for a summary with supporting participant quotes.

**TABLE 4:** Communication techniques identified from the included studies.

Themes	Examples of supporting statements
Technique (Theme): Ensuring conversation is individual and person‐centred	
Getting to know the person Identify communication needs beforehand Individualism Offers opportunity for person to express opinion Person‐centred Adaptive strategies and tools Appropriate for individual e.g. methodology, consent process, resources, environment	*‘This initial visit was also important in allowing the researcher to obtain background information about the person with dementia and their family carer and offered time for the researcher and participants to get to know each other’* (Murphy et al., 2010, p. 18). *‘It is so difficult to tell [my wife] what I think when I cannot remember the words, the pictures could help me a lot*’. (Murphy et al., 2010, p. 23; Murphy & Oliver, 2013, p. 177; Oliver et al., 2010, p. 30). *‘The interviewer should be prepared to change the images used in the symbols if an individual with dementia misinterprets the picture’* (Murphy et al., 2007a, p. 19). *‘Visual aids and assistance from person‐centred, empathic staff, maximised responses from residents’* (Godwin, 2014). *‘I considered this individual approach likely to be more successful than trying to engage people with severe levels of dementia (and some of whom would probably be hearing impaired) in a group discussion’* (Godwin, 2014, p. 104). *‘It must be emphasised that communication is a two‐way process. Consequently, staff must be given the time, skills, and motivation to talk with people with dementia, to record their views and to feed these back into everyday living choices and care plans’* (Murphy et al., 2007a, p. 63, 2007b). *‘In an effort to reduce cognitive load with preference rating, the PELI‐NH Likert scale (see Introduction) was reduced from five options to three: Very Important, Somewhat Important, and Not Important. Categorising preferences on a 3‐point scale has been used successfully in previous studies examining preference assessment in dementia’* (Burshnic & Bourgeois, 2022, p. 652).
Technique (Theme): Managing external influences	
Assumptions made of people with dementia Social hierarchy (reduced personhood or value) ‐ Towards people with dementia Social hierarchy (reduced personhood or value) ‐ Towards staff Social or infrastructure influences Using valid method of communication Implications of not communicating with people with dementia Challenges of including people with moderate‐to‐severe dementia in research e.g. ethical and consenting considerations Negative involvement of family during conversation Negative Involvement of others (care staff, care managers, researchers, other residents) during conversation Negative role of family regarding conversation/interview Negative role of others (health staff, managers) regarding conversation/interview Challenge of implementing research or new practice	‘*Despite low managerial expectations, all* [people with dementia] *were consulted and a majority expressed an opinion’* (Godwin, 2014, p. 112). ‘*It would be nice if a lot of people had more understanding and appreciate what you have got…like I was in town the other Saturday and this other lady started laughing because the way I was trying to struggle to talk and that started to make me feel uncomfortable and I thought if only she understood, then perhaps she would not stand there and laugh’* (Williamson, 2010, p. 21). *‘How another person reacts to you can make you very unhappy’* (Williamson, 2010, p. 17).
Technique (Theme): Engaging others	
Positive role of family prior to conversation Positive role of others (health staff, managers) prior to conversation Positive involvement of family during conversation Positive involvement of others (care staff, care managers, researchers, other residents) during conversation Use of dementia advisory group/patient and public involvement	*‘One* [participant] *did not say any words during the first interview. Prescription development included chart review for any clues to his communication abilities, consultation with the staff about demographic information, and likes and dislikes*’ (Acton et al., 2007, p. 42). ‘*Family members approved all symbols and often provided photographs or other materials for the 2D and 3D symbols’* (Fried‐Oken et al., 2012, p. 222). ‘*We could use it* [Talking Mats] *with the grandchildren, like a game but one that I can play too’*. (Murphy et al., 2010, p. 23). ‘…*unhappy to be left alone with the researcher who stopped the interview after one topic. The remaining three Talking Mats topics (and the two conversation interviews) were conducted with her family present to reassure her’* (Murphy et al., 2007a, p. 29). *‘The researchers selected photographs from Google Images™ and validated the stimuli by interviewing a panel of five older adults (3 females; 2 males), residing in an assisted living community’* (Burshnic & Bourgeois, 2022, p. 652).
Technique (Theme): Creating a structure to the conversation	
Conversation including based around topics or themes Structured conversation including reconfirmation, order by complexity	*‘…a greeting, introduction to the topic, introduction to the* [Augmentative and Alternative Communication] *device (if present), posing of questions and comments to prompt conversation about the selected topic, and closing grammar’* (Fried‐Oken et al., 2012, p. 223). *‘Talking Mats framework allowed them time and space to have their say, and helped to organise and structure their conversation with the person with dementia for whom they cared’* (Murphy et al., 2010, p. 38). *‘At the start of each interview, the researcher read from a script to ensure administration fidelity. The script informed residents of the purpose and procedures of the interview’* (Burshnic & Bourgeois, 2022, p. 652).
Technique (Theme): Facilitating the conversation	
Acknowledging participant characteristics Audio and voice recording Communication styles Experience and confidence is required Importance of providing time Need for innovative approaches Non‐verbal communication Reminiscence Role of observation Use of empathy Verbal cues of communication	‘*I think it would be nice if people gave you the courtesy of time to finish what you are trying to say… so communicating I think it is very important but* [I] *think it is nice if people give the benefit’* (Williamson, 2010, p. 20). ‘*care assistants involved in the research and I would use observation of body language to supplement our interpretation of any speech’* (Godwin, 2014, p. 106). ‘*Using visual aids, observation, activity, and non‐verbal communication achieved higher than expected participation’* (Godwin, 2014, p. 115). *‘Interviews were audio recorded with a digital recording device (Olympus WS‐852) to collect data on what residents said in response to preference questions’* (Burshnic & Bourgeois, 2022, p. 651). *‘When assessing understanding, it is important to take into account both verbal (speech and other vocalisations) and nonverbal (eye contact, gesture, facial expression and body posture) responses’* (Murphy et al., 2007a, p. 31). *‘Talking Mats clearly encouraged the participants with dementia to engage more in the discussions about managing daily living. This was demonstrated by changes in body language and the level of interaction with family carers and the researcher’* (Murphy et al., 2010, p. 28). *‘If the resident acknowledged the preference (*e.g. *“I enjoy that.”), but did not provide a rating, the researcher inquired further (*e.g. *“Is it very, somewhat or not important?”) while pointing to the list or sorting mat’* (Burshnic & Bourgeois, 2022, p. 653).

#### 3.5.1 Ensuring communication is individual and person‐centred

All authors emphasised the importance of focusing on the individual and being person‐centred. Getting to know the person prior to holding a research interview facilitates and develops two‐way interactions (Murphy et al., 2007a). This can be achieved by using personal and relevant images and symbols (Fried‐Oken et al., 2012). Other techniques include gathering information prior to a research interview by observing the person (Godwin, 2014) and/or talking to family and staff carers (Fried‐Oken et al., 2012; Godwin, 2014; Murphy et al., 2007a; Murphy, Gray, et al., 2010), and ensuring all methods and resources are appropriate to the individual's age and background (Fried‐Oken et al., 2012). Adaptations to pictures, symbols or sounds are needed to overcome visual, physical or cognitive challenges (Fried‐Oken et al., 2012; Godwin, 2014; Murphy et al., 2007a; Williamson, 2010); see Table 3. Limiting the number of options or choices presented can also be beneficial (Burshnic & Bourgeois, 2022) and researchers can start with general questions and follow up with more specific questions or prompts to confirm the answer. Ensuring that the research questions relate to topics the participant finds interesting is important for securing engagement (Fried‐Oken et al., 2012).

#### 3.5.2 Managing external influences

Social or external influences can affect the outcome of a research interview. Making assumptions about people should be avoided (Williamson, 2010) and any preconceptions should be acknowledged and addressed (Godwin, 2014). For example, where residents with dementia participated in a consultation on potential colour schemes within the care home, almost all contributed, contrary to the expectation of managers that only 25% of residents would provide appropriate responses, but because the result of the vote went against management wishes, the residents were overruled. This demonstrates both the under‐acknowledged potential of people with dementia to express opinions on topics which have significance for them and the way in which the assumptions and preferences of others can impact on these opinions being sought and implemented (Godwin, 2014). More subtle and implicit influences could also affect how participants responded. For example, one participant became increasingly agitated during a research interview because a member of the care home staff withheld his cigarettes, leading to the interview being terminated early (Murphy et al., 2007a).

#### 3.5.3 Engaging others

Engaging others in supporting the research can help facilitate the research process generally and communication during research interviews in particular. In one study, dementia service providers, care home staff and family members assisted with recruitment and consent processes (Murphy et al., 2007a) and in another they provided personal resources such as family photographs (Fried‐Oken et al., 2012). Burshnic and Bourgeois (2022) had generic photographs selected by researchers validated by a panel of five older people to ensure that each photograph was congruent with the preference being assessed. Advisory groups supported project design in three studies (Murphy et al., 2007a; Murphy et al., 2010; Williamson, 2010), adding robustness to research outcomes; see Table S2. Family members or care staff can offer comfort and familiarity if present during research interviews (Godwin, 2014; Murphy et al., 2007a; Williamson, 2010). However, such situations need to be handled sensitively to avoid family members or staff dominating the interview.

#### 3.5.4 Creating a structure for the interview

A clear structure enables participants to follow a conversation and, from a research point of view, enhances consistency in reporting (Fried‐Oken et al., 2012). Structuring the conversation during the research interview could involve for example a greeting, an introduction and familiarisation with topics and tools, conversational questions and/or prompts and concluding statements (Fried‐Oken et al., 2012). In this approach, consideration should be given to facilitating communication throughout the interview and not just in the section where the specific research questions are addressed Williamson (2010). Having research questions based around topics with which the participant is comfortable should support engagement (Fried‐Oken et al., 2012). Practice conversations could be used to develop the interview structure (Acton et al., 2007; Burshnic & Bourgeois, 2022). Communication, however, should be flexible and fluid, allowing for breaks when required (Acton et al., 2007; Fried‐Oken et al., 2012). It is important to be flexible with time allocation, to adjust this as necessary and to know when to terminate the interview (Murphy et al., 2007a).

#### 3.5.5 Facilitation skills

The skills considered important for facilitating research communication included the ability to demonstrate empathy (Godwin, 2014) and support dignity (Murphy, Gray, et al., 2010). Researchers need to observe carefully (Godwin, 2014), recognise participants' characteristics and behaviour, identify and interpret both verbal (Fried‐Oken et al., 2012; Murphy et al., 2007a) and non‐verbal (Burshnic & Bourgeois, 2022; Godwin, 2014; Murphy et al., 2007a; Murphy, Gray, et al., 2010) communications and adapt the interview accordingly.

While verbal recordings of interviews allow for detailed interpretation, analysis and validation of conversation outcomes (Acton et al., 2007; Burshnic & Bourgeois, 2022; Fried‐Oken et al., 2012; Murphy et al., 2007a; Murphy, Gray, et al., 2010; Williamson, 2010), the use of video recordings provides added benefits by allowing identification and analysis of non‐verbal communication (Acton et al., 2007; Fried‐Oken et al., 2012; Murphy et al., 2007a; Murphy et al., 2010), but must be used non‐invasively.

## 4 DISCUSSION

To our knowledge this is the first review exploring tools, methods and techniques used to support inclusion of people with moderate‐to‐severe dementia in research by eliciting their views, perspectives and preferences. This review identified several tools: Talking Mats, AAC Flexiboard, a preference sorting template with captioned generic photographs, development of personalised communication strategies and a consultation ballot. Thematic synthesis identified a set of five skills and techniques employed in the research to support the use of the specific tools and which researchers can draw upon in planning and conducting research interviews: ensuring conversations are individual and person‐centred, managing external influences, engaging others, creating structure and facilitation skills.

Research including people with moderate‐to‐severe dementia has tended to concentrate on communication practices that stimulate general conversation, increase engagement and social interaction (Swan et al., 2018). This review goes beyond this to identify tools, methods and techniques that could be utilised to elicit the views, perspectives and preferences of people with moderate‐to‐severe dementia in research studies. The tools identified were mostly based on using pictures and symbols, often accompanied by textual captions or labels, to augment communication (Burshnic & Bourgeois, 2022; Godwin, 2014; Murphy et al., 2007a; Murphy, Gray, et al., 2010; Murphy & Oliver, 2013; Oliver et al., 2010; Williamson, 2010). Images can be effective external memory aids (Phillipson & Hammond, 2018) and, with care, can convey complex concepts such as quality of life and elicit personal opinions (Bilodeau et al., 2019; Collins et al., 2022; Williamson, 2010). However, pictures and symbols need to be clear and free from confusion or misinterpretation and should not appear childish (Collins et al., 2022; Mackenzie et al., 2011). One way to address some of these challenges is to use, where available, suitable personal pictures, photographs or objects (Bayles & Tomoeda, 2014; Fried‐Oken et al., 2012), which may require less cognitive effort (Brandão et al., 2014; McKelvey et al., 2010). Indeed, Burshnic and Bourgeois (2022) suggested that personally relevant images would have encouraged more in‐depth conversations than the generic photographs used in their study. However, it may be difficult to obtain suitable personal pictures or objects, and this might not always be feasible in a research context.

The picture‐based Talking Mats and AAC Flexiboard tools were considered effective, but with limitations (Fried‐Oken et al., 2012; Murphy et al., 2007a; Murphy, Gray, et al., 2010; Murphy & Oliver, 2013). They may not be appropriate for some (Macer et al., 2009; McKillop & Wilkinson, 2004) and effectiveness for people with more severe dementia remains unclear (Collins et al., 2022). It could be argued that if people with moderate‐to‐severe dementia need to be trained to use these tools, a simpler tool should be designed. Development of personalised communication strategies to use in interviews appears promising (Acton et al., 2007) but more evidence is needed regarding effectiveness. It is important to acknowledge the resources required to adopt these kinds of methods in research studies, and the challenges of gathering information from samples large enough to yield reliable data.

While the review demonstrates that various tools, methods and techniques, often including the use of pictures and symbols, can be used to facilitate and stimulate conversations in research interviews, the evidence‐base is limited. It does not provide strong evidence for any of the specific tools reviewed, and nor does it provide a basis for recommending any one tool. Each tool has advantages and disadvantages, and its suitability in any given situation may depend on several factors, including the abilities of the participants, capability and training of the researchers and cost.

Alongside reviewing specific tools, we have drawn together knowledge about the skills and techniques that can be useful for facilitating communication with people who have moderate‐to‐severe dementia, and these may also be relevant for other groups of people where effective communication presents challenges. This offers useful guidance for researchers and practitioners which is supported by evidence from other sources. First, taking time to get to know the person, build rapport and set up as much of a two‐way exchange as possible is essential (Alsawy et al., 2020; Brooker & Latham, 2015; Clare et al., 2008b; Collins et al., 2022; Williams et al., 2020). Second, researchers must acknowledge and avoid assumptions and manage any possible influences that could have a negative effect on communication. Researchers may need to overcome negative expectations held by others in the person's environment who may not understand the potential for participation and who may assume the person is unable to contribute and feel they are protecting the person from something that is too challenging. Third, enabling positive relationships between the participant and others can facilitate conversations (Alsawy et al., 2017; Wray, 2020). People with moderate‐to‐severe dementia may appreciate the guidance and security others provide, as long as this does not limit the person's ability to offer views and opinions (Alsawy et al., 2020). Fourth, having a structure, particularly using themes based on topics familiar to the person with dementia at the present time, helps facilitate and focus the research interview (Wray, 2020). Finally, researchers need to acquire and employ facilitation skills to aid conversation flow, respond appropriately to participants' needs and wishes, allow sufficient time for participants to respond and have the understanding and confidence to delay or terminate the interview. Skills in understanding and interpreting both verbal and non‐verbal communications are vital. Evidence from sources outside this review suggests that without sufficient proficiency, communication attempts can be misinterpreted as murmurings or ramblings, and non‐verbal cues may be missed, and hence care should be taken to avoid these pitfalls (Clare et al., 2012; Clare, Quinn et al., 2014; Hughes, 2013; Quinn et al., 2014; Round et al., 2014).

While researchers may face challenges in recruiting people with moderate‐to‐severe dementia, securing informed consent and finding effective ways to elicit their views, the inclusion of people with moderate‐to‐severe dementia in the studies covered by this review demonstrates that it is feasible. With appropriate support, people with moderate‐to‐severe dementia can engage in the research process (Bilodeau et al., 2019) and their opinions and views can be included (Mann & Hung, 2019; Ries et al., 2017). Including their perspective in research will enrich understanding of their preferences and needs and the resulting evidence will better equip carers, practitioners and service providers to meet these needs, with potential to enhance well‐being and quality of life.

### 4.1 Limitations

This review has some limitations. One important consideration is the difficulty of determining from study reports a clear understanding of the stage or severity of dementia among the participants. Dementia stage and severity were reported in various ways and sometimes in the form of subjective evaluations. Therefore, it is not possible to precisely specify the cognitive ability of all participants. In addition, some studies included a small number of people with mild dementia, presenting challenges for interpretation of results.

Because of our focus on tools that have been assessed for use with people who have moderate‐to‐severe dementia, we may have missed other tools that, while designed for people with mild or more severe dementia, could be suitable for people with moderate‐to‐severe dementia, either in their current form or following adaptation. Some communication tools designed for people with early‐stage dementia may be suitable for people with moderate‐to‐severe dementia, but as their use with people with more severe dementia has not been reported, they were not included here.

We did not exclude studies based on quality. Study quality was generally only moderate, reflecting the exploratory nature and limited scope of the studies and the small sample sizes. Future research using more rigorous designs may yield more robust evidence. There was only one study scoring slightly lower than the rest that fell into the low quality range; this presented a distinct approach to capturing participants' perspectives rather than contributing to a wider evaluation of a given tool, and hence its retention did not affect the evaluation of other approaches.

## 5 CONCLUSIONS AND IMPLICATIONS

This review has examined currently available tools and methods for facilitating communication that could support the inclusion of people with moderate‐to‐severe dementia in research and enabling them to contribute their opinions and views. We identified a small number of structured tools, mostly based on using pictures and symbols to augment communication, designed to facilitate and stimulate conversation during research interviews. The study designs, tools and outcome measures were heterogeneous including variability in interview content, duration and frequency. Combined with the variability in quality, limited sample sizes and limited confidence in statistical analysis of the studies, outcomes or conclusions, evidence was limited and there was no basis for recommending any one specific tool. All the tools have some potential utility, and researchers may select a specific approach based on the context, on the capabilities of researchers and participants, and on the resources available. Regardless of the specific communication method used, it is vital to ensure that participation in research is tailored to individual capabilities and needs. We also identified a set of general skills and techniques that researchers can employ to support inclusion of this group in research, and that are also highly relevant for practitioners. By deploying these skills when engaging with people who have mild‐to‐moderate dementia, researchers can promote engagement and ensure the experience of participation is positive.

## AUTHOR CONTRIBUTIONS

Rachel Collins: Full‐text screening, data extraction, assessment of study quality, planning and conducting analyses, interpretation of results and drafting review text. Anthony Martyr: Data extraction, assessment of study quality, planning and conducting analyses, interpretation of results and drafting review text. Anna Hunt: Developing and piloting search strategy, conducting searches, title screening, abstract screening, full‐text screening, data extraction, assessment of study quality, planning and conducting analyses, interpretation of results and drafting review text. Catherine Quinn: Developing and piloting search strategy, conducting searches, title screening, abstract screening and drafting review text. Claire Pentecost: Drafting review text. Julian C. Hughes: Developing search strategy and drafting review text. Linda Clare: Developing search strategy, full‐text screening and drafting review text. All authors contributed to the critical revision of the article and approved the version to be published.

## FUNDING INFORMATION

‘Improving the experience of Dementia and Enhancing Active Life: living well with dementia. The IDEAL study’ was funded jointly by the Economic and Social Research Council (ESRC) and the National Institute for Health and Care Research (NIHR) through grant ES/L001853/2. Investigators: L. Clare, I.R. Jones, C. Victor, J.V. Hindle, R.W. Jones, M. Knapp, M. Kopelman, R. Litherland, A. Martyr, F.E. Matthews, R.G. Morris, S.M. Nelis, J.A. Pickett, C. Quinn, J. Rusted and J. Thom. ESRC is part of UK Research and Innovation (UKRI). ‘Improving the experience of Dementia and Enhancing Active Life: a longitudinal perspective on living well with dementia. The IDEAL‐2 study’ is funded by Alzheimer's Society, grant number 348, AS‐PR2‐16‐001. Investigators: L. Clare, I.R. Jones, C. Victor, C. Ballard, A. Hillman, J.V. Hindle, J. C. Hughes, R.W. Jones, M. Knapp, R. Litherland, A. Martyr, F.E. Matthews, R.G. Morris, S.M. Nelis, C. Quinn and J. Rusted. This report is independent research supported by the National Institute for Health and Care Research Applied Research Collaboration South West Peninsula. The views expressed in this publication are those of the authors and not necessarily those of the ESRC, UKRI, NIHR, the Department of Health and Social Care, the National Health Service or Alzheimer's Society. The support of ESRC, NIHR and Alzheimer's Society is gratefully acknowledged. For the purposes of open access, the authors have applied a Creative Commons Attribution (CC BY) licence to any Author Accepted Manuscript version arising.

## CONFLICT OF INTEREST STATEMENT

The authors have no conflicts of interest to report.

## Supporting information


Data S1.

## Data Availability

The data that support the findings of this study are available from the corresponding author upon reasonable request.
