# Supplementary material for: Methods and approaches to facilitate inclusion of the views, perspectives and preferences of people with moderate‐to‐severe dementia in research: A narrative systematic review
Source: Int J Older People Nurs. 2023 Dec 11;19(1):e12594. doi: 10.1111/opn.12594 (PMC11475515; doi:10.1111/opn.12594)
Supplement: Supplementary file 1 — Data S1. [file OPN-19-e12594-s001.docx]

**Supplementary Material**

**Search strings used in the review**

PubMed

((dementia[Title] OR alzheimer*[Title] OR vascular dementia[Title] OR lewy body[Title] OR frontotemporal[Title]) AND (nonverbal[Title/Abstract] OR communication[Title/Abstract] OR communicate[Title/Abstract] OR language[Title/Abstract] OR speech[Title/Abstract] OR speak[Title/Abstract] OR voice[Title/Abstract] OR talk*[Title/Abstract] OR talking mats[Title/Abstract] OR Augment* Alternative[Title/Abstract] OR AAC[Title/Abstract])) AND (English[Language]) AND "Humans"[Mesh]

AgeLine

TI ( dementia or alzheimer* OR vascular dementia OR lewy body OR frontotemporal ) AND AB ( nonverbal OR communication OR communicate OR language OR speech OR speak OR voice OR talk* OR talking mats OR Augment* Alternative OR AAC )

CINAHL

TI ( dementia or alzheimer* or vascular dementia or lewy body or frontotemporal ) AND AB ( nonverbal or communication or communicate or language or speech or speak or voice or talk* or talking mats or Augment* Alternative or AAC ) AND LA English

Embase, PsycINFO, and Social Policy and Practice (all via OVID)

1 (dementia or alzheimer* or vascular dementia or lewy body or frontotemporal).ti. and (nonverbal or communication or communicate or language or speech or speak or voice or talk* or talking mats or Augment* Alternative or AAC).ab. and English.lg.

limit 1 to human

Web of Science

dementia or alzheimer* or vascular dementia or lewy body or frontotemporal (Title) and nonverbal OR communication OR communicate OR language OR speech OR speak OR voice OR talk* OR talking mats OR Augment* Alternative OR AAC (Abstract) and English (Language)

Supplementary Table 1. Articles excluded at the full-text screening stage

| Reason for exclusion | Study name |
| --- | --- |
| Not a specific communication tool (n=10) | [Bilodeau et al. (2019)](#_ENREF_3); [Branco et al. (2015)](#_ENREF_5); [Mayhew et al. (2001)](#_ENREF_13); [Mitchell and Koch (1997)](#_ENREF_14); [Reid et al. (2001)](#_ENREF_22); [Stara et al. (2021)](#_ENREF_25); [Tappen et al. (1997)](#_ENREF_27); [Tetley (2013)](#_ENREF_28); [Varela Suárez (2018)](#_ENREF_31); [Wied et al. (2021)](#_ENREF_32) |
| Not eliciting views or perspectives (n=2) | [Bourgeois et al. (2001)](#_ENREF_4); [Mabire et al. (2022)](#_ENREF_11) |
| No new data (n=2) | [Acton et al. (1999)](#_ENREF_1); [Thompson (2002)](#_ENREF_29) |
| Carers or care staff only interviewed (n=2) | [Mason-Baughman and Lander (2012)](#_ENREF_12); [Stanyon et al. (2016)](#_ENREF_24) |
| No indication of severity (n=6) | [Goh et al. (2022)](#_ENREF_9); [Law and Ashworth (2022)](#_ENREF_10); [Murphy and Ewing (2018)](#_ENREF_15); [Reitz and Dalemans (2016)](#_ENREF_23); [Stevenson and Taylor (2019)](#_ENREF_26); [Towsley et al. (2021)](#_ENREF_30) |

**Supplementary Table 2.** Quality assessment (QATSDD) **scores for the included studies**

| QATSDD Statement | [Acton et al. (2007)](#_ENREF_2) | [Burshnic and Bourgeois (2022)](#_ENREF_6) | [Fried-Oken et al. (2012)](#_ENREF_7) | [Godwin (2014)](#_ENREF_8) | [Murphy et al. (2007a)](#_ENREF_16); [Murphy et al. (2007b)](#_ENREF_17); [Murphy, Gray, et al. (2010)](#_ENREF_18) | [Murphy and Oliver (2013)](#_ENREF_19); [Murphy, Oliver and Cox (2010)](#_ENREF_20); [Oliver et al. (2010)](#_ENREF_21) | [Williamson (2010)](#_ENREF_33) |
| --- | --- | --- | --- | --- | --- | --- | --- |
| 1. Explicit theoretical framework | 3 | 0 | 0 | 2 | 0 | 0 | 0 |
| 2. Statement of aims/objectives in main body of report | 3 | 3 | 3 | 3 | 3 | 2 | 3 |
| 3. Clear description of research setting | 3 | 3 | 2 | 2 | 3 | 3 | 2 |
| 4. Evidence of sample size considered in terms of analysis | 0 | 0 | 0 | 0 | 0 | 0 | 0 |
| 5. Representative sample of target group of a reasonable size | 1 | 1 | 1 | 1 | 1 | 1 | 1 |
| 6. Description of procedure for data collection | 3 | 3 | 3 | 2 | 3 | 3 | 2 |
| 7. Rationale for choice of data collection tool(s) | 1 | 1 | 3 | 2 | 3 | 3 | 2 |
| 8. Detailed recruitment | 0 | 2 | 2 | 2 | 3 | 3 | 2 |
| 9. Statistical assessment of reliability and validity of measurement tool(s) (Quantitative only) | 0 | 2 | 0 | 0 | 1 | 0 | 2 |
| 10. Fit between stated research question and method of data collection (Quantitative) | 3 | 3 | 2 | 2 | 3 | 3 | 3 |
| 11. Fit between stated research question and format and content of data collection tool e.g., interview schedule (Qualitative) | 2 | 2 | 1 | 1 | 3 | 3 | 2 |
| 12. Fit between research question and method of analysis | 2 | 3 | 2 | 1 | 3 | 3 | 2 |
| 13. Good justification for analytical method selected | 1 | 0 | 1 | 0 | 2 | 3 | 0 |
| 14. Assessment of reliability of analytical process (Qualitative only) | 2 | 2 | 2 | 0 | 3 | 3 | 0 |
| 15. Evidence of user involvement in design | 0 | 2 | 1 | 1 | 3 | 3 | 2 |
| 16. Strengths and limitations critically discussed | 2 | 3 | 2 | 1 | 1 | 1 | 3 |
| Total Score (out of 48) | 26 | 30 | 25 | 20 | 35 | 34 | 26 |
| Percentage | 54.2 | 62.5 | 52.1 | 41.7 | 72.9 | 70.8 | 54.2 |

Note: 0 = not at all, 1 = very slightly, 2 = moderately, 3 = complete. As all reviewed articles used both quantitative and qualitative methods, all 16 items were rated for each article.

**Supplementary references**

Acton, G. J., Mayhew, P. A., Hopkins, B. A., & Yauk, S. (1999). Communicating with individuals with dementia. The impaired person's perspective. *Journal of Gerontological Nursing*, *25*(2), 6-13. <https://doi.org/10.3928/0098-9134-19990201-04>

Acton, G. J., Yauk, S., Hopkins, B. A., & Mayhew, P. A. (2007). Increasing social communication in persons with dementia. *Research and Theory for Nursing Practice*, *21*(1), 32-44. <https://doi.org/10.1891/rtnpij-v21i1a005>

Bilodeau, G., Witteman, H., Légaré, F., Lafontaine-Bruneau, J., Voyer, P., Kröger, E., Tremblay, M. C., & Giguere, A. M. C. (2019). Reducing complexity of patient decision aids for community-based older adults with dementia and their caregivers: multiple case study of Decision Boxes. *BMJ Open*, *9*(5), e027727. <https://doi.org/10.1136/bmjopen-2018-027727>

Bourgeois, M. S., Dijkstra, K., Burgio, L., & Allen-Burge, R. (2001). Memory aids as an augmentative and alternative communication strategy for nursing home residents with dementia. *Augmentative and Alternative Communication*, *17*(3), 196-210. <https://doi.org/10.1080/714043383>

Branco, R. M., Quental, J., & Ribeiro, O. (2015). Getting closer, empathising and understanding: setting the stage for a codesign project with people with dementia. *Interaction Design and Architectures*(26), 114-131. <https://doi.org/10.55612/s-5002-026-007>

Burshnic, V. L., & Bourgeois, M. S. (2022). A seat at the table: supporting persons with severe dementia in communicating their preferences. *Clinical Gerontologist*, *45*(3), 647-660. <https://doi.org/10.1080/07317115.2020.1764686>

Fried-Oken, M., Rowland, C., Daniels, D., Dixon, M., Fuller, B., Mills, C., Noethe, G., Small, J., Still, K., & Oken, B. (2012). AAC to support conversation in persons with moderate Alzheimer’s disease. *Augmentative and Alternative Communication*, *28*(4), 219-231. <https://doi.org/10.3109/07434618.2012.732610>

Godwin, B. (2014). Colour consultation with dementia home residents and staff. *Quality in Ageing and Older Adults*, *15*(2), 102-119. <https://doi.org/10.1108/qaoa-04-2013-0006>

Goh, A. M. Y., Polacsek, M., Malta, S., Doyle, C., Hallam, B., Gahan, L., Low, L. F., Cooper, C., Livingston, G., Panayiotou, A., Loi, S. M., Omori, M., Savvas, S., Burton, J., Ames, D., Scherer, S. C., Chau, N., Roberts, S., Winbolt, M., . . . Dow, B. (2022). What constitutes 'good' home care for people with dementia? An investigation of the views of home care service recipients and providers. *BMC Geriatrics*, *22*(1), Article 42. <https://doi.org/10.1186/s12877-021-02727-4>

Law, E., & Ashworth, R. (2022). Facilitators and barriers to research participation in care homes: thematic analysis of interviews with researchers, staff, residents and residents’ families. *Journal of Long-Term Care*, *2022*(1), 49-60. <https://doi.org/10.31389/jltc.87>

Mabire, J.-B., Gay, M.-C., Charras, K., & Vernooij-Dassen, M. (2022). Impact of a psychosocial intervention on social interactions between people with dementia: an observational study in a nursing home. *Activities, Adaptation & Aging*, *46*(1), 73-89. <https://doi.org/10.1080/01924788.2021.1966574>

Mason-Baughman, M. B., & Lander, A. (2012). Communication strategy training for caregivers of individuals with dementia. *Perspectives on Gerontology*, *17*(3), 78-83. <https://doi.org/10.1044/gero17.3.78>

Mayhew, P. A., Acton, G. J., Yauk, S., & Hopkins, B. A. (2001). Communication from individuals with advanced DAT: can it provide clues to their sense of self-awareness and well-being? *Geriatric Nursing*, *22*(2), 106-110. <https://doi.org/10.1067/mgn.2001.115198>

Mitchell, P., & Koch, T. (1997). An attempt to give nursing home residents a voice in the quality improvement process: the challenge of frailty. *Journal of Clinical Nursing*, *6*(6), 453-461. <https://doi.org/10.1111/j.1365-2702.1997.tb00342.x>

Murphy, J., & Ewing, N. (2018). *Self-managing long term conditions smartly: final report incorporating all 3 reports to funder at 6, 12 and 18 months.* Final Report July 2018 - Incorporating all 3 reports to the Health and Social Care ALLIANCE Scotland <https://www.talkingmats.com/wp-content/uploads/2018/07/20180717-Alliance-full-report.pdf>

Murphy, J., Gray, C. M., & Cox, S. (2007a). *Communication and dementia: how Talking Mats can help people with dementia to express themselves*. Project Report. Joseph Rowntree Foundation. <https://eprints.gla.ac.uk/61498/1/61498.pdf>

Murphy, J., Gray, C. M., & Cox, S. (2007b). The use of Talking Mats to improve communication and quality of care for people with dementia. *Housing, Care and Support*, *10*(3), 21-28. <https://doi.org/10.1108/14608790200700018>

Murphy, J., Gray, C. M., van Achterberg, T., Wyke, S., & Cox, S. (2010). The effectiveness of the Talking Mats framework in helping people with dementia to express their views on well-being. *Dementia*, *9*(4), 454-472. <https://doi.org/10.1177/1471301210381776>

Murphy, J., & Oliver, T. (2013). The use of Talking Mats to support people with dementia and their carers to make decisions together. *Health & Social Care in the Community*, *21*(2), 171-180. <https://doi.org/10.1111/hsc.12005>

Murphy, J., Oliver, T., & Cox, S. (2010). *Talking Mats® and involvement in decision making for people with dementia and family carers*. Joseph Rowntree Foundation.

Oliver, T., Murphy, J., & Cox, S. (2010). ‘She can see how much I actually do!’ Talking Mats®: helping people with dementia and family carers to discuss managing daily living. *Housing, Care and Support*, *13*(3), 27-35. <https://doi.org/10.5042/hcs.2010.0708>

Reid, D., Ryan, T., & Enderby, P. (2001). What does it mean to listen to people with dementia? *Disability & Society*, *16*(3), 377-392. <https://doi.org/10.1080/09687590120045941>

Reitz, C., & Dalemans, R. (2016). The use of 'Talking Mats' by persons with Alzheimer in the Netherlands: increasing shared decision-making by using a low-tech communication aid. *Journal of Social Inclusion*, *7*(2), 35-47. <https://doi.org/10.36251/josi.110>

Stanyon, M. R., Griffiths, A., Thomas, S. A., & Gordon, A. L. (2016). The facilitators of communication with people with dementia in a care setting: an interview study with healthcare workers. *Age & Ageing*, *45*(1), 164-170. <https://doi.org/10.1093/ageing/afv161>

Stara, V., Vera, B., Bolliger, D., Rossi, L., Felici, E., Di Rosa, M., de Jong, M., & Paolini, S. (2021). Usability and acceptance of the embodied conversational agent Anne by people with dementia and their caregivers: exploratory study in home environment settings. *JMIR mHealth and uHealth*, *9*(6), e25891. <https://doi.org/10.2196/25891>

Stevenson, M., & Taylor, B. J. (2019). Involving individuals with dementia as co-researchers in analysis of findings from a qualitative study. *Dementia*, *18*(2), 701-712. <https://doi.org/10.1177/1471301217690904>

Tappen, R. M., Williams-Burgess, C., Edelstein, J., Touhy, T., & Fishman, S. (1997). Communicating with individuals with Alzheimer's disease: examination of recommended strategies. *Archives of Psychiatric Nursing*, *11*(5), 249-256. <https://doi.org/10.1016/s0883-9417(97)80015-5>

Tetley, J. (2013). Articulation, service use, managing and coping: understanding the needs of older people and carers living with dementia. *International Practice Development Journal*, *3*(2), Article 1.

Thompson, P. M. (2002). Communicating with dementia patients on hospice. *American Journal of Alzheimer's Disease and Other Dementias*, *17*(5), 299-302. <https://doi.org/10.1177/153331750201700513>

Towsley, G. L., Wong, B., Baier, R. R., & Neller, S. (2021). An efficacy trial of long-term care residents with Alzheimer's disease using videos to communicate care preferences. *Journal of the American Medical Directors Association*, *22*(7), 1559-1560. <https://doi.org/10.1016/j.jamda.2021.02.032>

Varela Suárez, A. (2018). The question‐answer adjacency pair in dementia discourse. *International Journal of Applied Linguistics*, *28*(1), 86-101. <https://doi.org/10.1111/ijal.12185>

Wied, T. S., Haberstroh, J., Gather, J., Karakaya, T., Oswald, F., Qubad, M., Scholten, M., Vollmann, J., Pantel, J., & the ENSURE Consortium. (2021). Supported decision-making in persons with dementia: development of an enhanced consent procedure for lumbar puncture. *Frontiers in Psychiatry*, *12*(1), Article 780276. <https://doi.org/10.3389/fpsyt.2021.780276>

Williamson, T. (2010). *My name is not dementia: people with dementia discuss quality of life indicators*. Alzheimer's Society.
